# Acute and Chronic Impact of Dynamic Exercise on Arterial Stiffness in Older Hypertensives

**DOI:** 10.2174/1874192400802010003

**Published:** 2008-02-12

**Authors:** Kunihiko Aizawa, Robert J. Petrella

**Affiliations:** 1 Aging, Rehabilitation, & Geriatric Care Research Centre of the Lawson Health Research Institute at Parkwood Hospi-tal; 2 School of Kinesiology; 3 Schulich School of Medicine and Dentistry, The University of Western Ontario, London, ON, Canada

**Keywords:** Hypertension, exercise, elderly, arterial stiffness

## Abstract

Arterial stiffness increases with ageing and hypertension. Regular physical activity has been recommended as an important management component of hypertension. The purpose of this study was to examine the acute impact of maximal dynamic exercise and the effect of 20 weeks of aerobic exercise on arterial stiffness of the carotid and brachial arteries in older hypertensives. Nine previously sedentary and treated older hypertensives (2 men and 7 women, age 68.2 ± 5.4 yrs) performed maximal treadmill exercise to volitional fatigue while arterial stiffness indices (arterial distensibility and β stiffness index) were measured prior to, immediately (about 10 min) following, and 24 h following maximal exercise. These measurements were repeated following 20 weeks of moderate intensity aerobic exercise training. Maximal exercise had no impact on arterial stiffness indices immediately and 24 h following exercise intervention. Following 20 weeks of training, arterial stiffness indices remained unchanged at rest and following maximal exercise. These data show that, in older hypertensives, 1) acute maximal dynamic exercise had no impact on arterial stiffness of the carotid and brachial arteries, and 2) 20 weeks of moderate intensity aerobic exercise training failed to modify arterial stiffness.

## INTRODUCTION

Hypertension is one of the leading risk factors for mortality in developed countries. It also serves as a risk factor for cardiovascular and kidney diseases such as stroke and renal failure. Despite the development of effective anti-hypertensive medications, pharmacological treatment for hypertension has been unable to make substantial impact on control rates [1]. Lifestyle modification, such as regular physical activity, has been performed to reduce cardiovascular disease risk burden and is recommended as initial management for hypertension [2].

Regular physical activity, such as aerobic exercise, has been shown to be associated with reduced arterial stiffness, a possible mechanism of initiation and/or progression of hypertension [3], of central arteries in cross-sectional [4-6] and intervention studies [5]. Moreover, although not all study documented [7], arterial stiffness of central and peripheral arteries has been shown to be reduced even 30~60 min following both sub-maximal and maximal dynamic exercise in young healthy subjects [8, 9]. With repeated exposures of reduced arterial smooth muscle tone by aerobic exercise training, this may be a possible mechanism of reduced arterial stiffness observed in previous studies [4-6]. In older hypertensives, however, the acute and chronic impact of dynamic exercise on arterial stiffness is not fully documented to date. Indeed, in contrast to favorable effect of aerobic exercise training on arterial stiffness in middle-aged and older healthy adults [5], arterial stiffness of central and peripheral arteries remained unchanged with 8 weeks of moderate intensity aerobic exercise training in older adults with isolated systolic hypertension [10]. Whether longer duration (>8 weeks) of aerobic exercise training would reduce arterial stiffness in older hypertensives has not been determined.

Therefore, this study examined the impact of acute maximal treadmill exercise on arterial stiffness indices, arterial distensibility and β stiffness index, of the carotid and brachial arteries in older hypertensives immediately following and 24 h following exercise intervention. In addition, this study also examined how 20 weeks of moderate intensity aerobic exercise training would influence on arterial stiffness indices at rest and following the acute maximal exercise. We hypothesized that 1) an acute maximal treadmill exercise would not reduce arterial stiffness indices at baseline but would reduce them following the 20 weeks of moderate intensity aerobic exercise training, and 2) 20 weeks of moderate intensity aerobic exercise training would reduce arterial stiffness indices at rest.

## METHODS

### Subjects

Nine older sedentary adults (two males and seven females) with treated stage 1 HT [11] who were over 60 years old were recruited. HT was defined as systolic blood pressure (SBP) > 140 but < 160 mmHg, diastolic blood pressure (DBP) > 90 but < 100 mmHg, or administration of antihypertensive drugs. All subjects took stable (> 1 year) anti-hypertensive medication(s) at time of enrollment and continued the treatment throughout the study period. Before participating in the study, all subjects received a full screening medical and physical examination to exclude persons with: unstable cardiovascular, renal, pulmonary or chronic musculoskeletal disorders preventing them from engaging exercise training program for twenty weeks. Four subjects took hydrochlorothiazide, three took an ACE inhibitor, two took an angiotensin II receptor blocker, and one each took a β-blocker and a calcium channel-blocking agent. The study was approved by the University of Western Ontario Research Ethics Board for Health Sciences Research Involving Human Subjects and all subjects gave written informed consent prior to participation.

### Study Design

In order to determine the acute effect of maximal exercise, arterial stiffness indices were assessed prior to, immediately (about 10 min) following, and 24 h following an acute maximal treadmill exercise. Arterial stiffness indices at rest were also assessed at 8 week of training and after the completion of the 20-week exercise training period. Subjects were encouraged to maintain their normal diet and other lifestyle habits throughout the study period. All measurements were performed in a quiet, temperature-controlled laboratory. Subjects reported to the laboratory after at least a 4-h fast including caffeine and were tested at the same time of day to minimize potential diurnal variations. In addition, subjects were studied at least 24 h after their last exercise session to minimize the acute effect of exercise on resting arterial stiffness indices [8, 9].

### Resting Blood Pressure

Following a 15-min supine rest, brachial BP [SBP,DBP,mean arterial pressure (MAP),pulse pressure (PP)] and heart rate (HR) were measured with an automated oscillometric device (HDI/PulseWave^TM^ CR-2000 research cardiovascular system, Hypertension Diagnostic Inc., Eagan, MN). All measurements were duplicated after a further 5-min rest.

### Arterial Stiffness of the Carotid and Brachial Arteries

Following BP measurements, the measurement of arterial stiffness indices in carotid and brachial arteries was performed using a 10 MHz linear array transducer attached to a high-resolution ultrasound machine (VingMed System 5, GE Ultrasound A/S, Horton, Norway). All scans were performed by the same investigator and made under similar conditions. The longitudinal 2D carotid internal diameter of the right common carotid artery 1-2 cm proximal to the carotid bifurcation was imaged with simultaneous measurements of BP in the left brachial artery. The longitudinal 2D brachial internal diameter on the right arm 3-5 cm proximal to the antecubital fossa was also imaged in a similar manner. In each image, the largest diameter, strong wall signals and longitudinal section of the artery were searched. Images were recorded to S-VHS tape for later offline analysis. HR was continuously monitored with a three-lead electrocardiogram. The images of carotid and brachial arteries were analyzed from the stored images using calipers with a resolution of 0.03 mm. All images were analyzed by the same investigator. Lumen diameter was defined as the distance between near-wall interface and far-wall interface. Time points that corresponded with systole (maximal expansion) and diastole (baseline) were selected. To characterize arterial stiffness, arterial distensibility (elastic response of the artery as a hollow structure) [12] and β stiffness index (stiffness of the arterial wall independent of distending pressure) [13] of the carotid and brachial arteries were calculated using the following equations: (Dsys^2^ – Ddia^2^)/PP·Ddia^2^ and ln(SBP/DBP)/[(Dsys – Ddia)/Ddia], where Dsys and Ddia are diameters at systole and diastole, respectively [14].

### Maximal Treadmill Exercise

Subjects performed a graded exercise treadmill test (Bruce protocol) to volitional fatigue. Their exercise capacity expressed as VO_2_max estimated from metabolic equivalents, and a target training HR were determined.

### Aerobic Exercise Training

Subjects were given a standard exercise training prescription based on recommendations of the American College of Sports Medicine [15] consisting of the following: exercise intensity set at a heart rate representing 70% VO_2_max; 30 or more min of exercise at prescribed intensity; exercise bouts on three or more days per week. Subjects performed aerobic exercise, such as cycle ergometry and/or treadmill walking, for 20 weeks at our laboratory and at their home. On average, subjects performed ~50% of their exercise session at the laboratory and 50% at their home. To ensure compliance, subjects were asked to keep a diary for recording the duration of training and maximum HR achieved.

### Statistics

Data are presented as means ± SD unless otherwise stated. The effect of acute maximal treadmill exercise was analyzed prior to, immediately following, and 24 h following maximal treadmill exercise using repeated measures of ANOVA. The effect of aerobic exercise training was also analyzed prior to, at 8 week, and following the completion of 20-week moderate intensity aerobic exercise training using repeated measures of ANOVA. As arterial distensibility is influenced by changes in MAP, analysis of covariance (ANCOVA) was performed with MAP as a covariate to determine the effect of an acute maximal treadmill exercise and 20 weeks of aerobic exercise training on arterial distensibility. All statistical analysis was done using SPSS (version12.0, SPSS Inc., Chicago, IL) and significance was set at p<0.05.

## RESULTS

Eight of nine subjects completed all the measurements and the 20 weeks of exercise training. Subjects exercised for an average of 4.3 ± 1.2 d/wk, 44.2 ± 13.6 min/d, and at 91.1 ± 6.1% of target HR. One subject did not complete the measurements at eight weeks and following the completion of exercise training due to a compassionate reason. There were no adverse events reported during the study period.

### Subject’s Characteristics

The subject characteristics are shown in Table **1**. No changes were observed in subject characteristics throughout the study period.

### Acute Impact of Maximal Treadmill Exercise on Arterial Stiffness Indices and BP

Table **2** shows the effect of acute maximal treadmill exercise on arterial distensibility and β stiffness index of the carotid and brachial arteries. There was no significant difference in arterial distensibility nor β stiffness index immediately following and 24 h following an acute maximal treadmill exercise compared to baseline. An ANCOVA was performed with HR as a covariate because HR was significantly changed immediately following acute maximal exercise compared to baseline and 24 h following an acute exercise (Table **3**). However, no significant difference was observed in neither arterial distensibility nor β stiffness index (data not shown). Acute maximal treadmill exercise lowered DBP and MAP (Table **3**; main effect of time, p<0.05) and it tended to lower SBP (main effect of time, p=0.09). PP remained unchanged.

### Effect of 20 Weeks of Aerobic Exercise Training on Arterial Stiffness Indices, BP, and Exercise Capacity

Fig. (**1**) and (**2**) show the effect of 20 weeks of aerobic exercise training on the arterial distensibility and β stiffness index of the carotid and brachial arteries, respectively. Neither arterial distensibility nor β stiffness index changed significantly following the 20 weeks of exercise training. DBP and MAP tended to be lower at 8 week and following the completion of exercise training, but these did not reach statistical significance (Table **4**; main effect of time, p=0.10). SBP, PP, and HR remained unchanged throughout the study period. Exercise capacity, expressed as VO_2_max, did not increase following 20 weeks of aerobic exercise training (Table **1**).

## DISCUSSION

In this study, the acute impact of maximal dynamic exercise and effect of 20 weeks of moderate intensity aerobic exercise training on arterial stiffness of the carotid (central) and brachial (peripheral) arteries in older hypertensives were studied. Contrary to previous studies in healthy young subjects that showed reduced arterial stiffness following sub-maximal and maximal dynamic exercise [8, 9], arterial stiffness in our older hypertensive subjects remained unchanged following acute maximal dynamic exercise. In addition, 20 weeks of aerobic exercise training was unable to modify arterial stiffness neither at rest nor following an acute maximal dynamic exercise.

### Acute Impact of Maximal Dynamic Exercise on Arterial Stiffness Indices

A reduction of arterial stiffness following an acute bout of dynamic exercise observed in previous studies [8, 9] is thought to be due to the relaxation of vascular smooth muscle tone, transferring wall stress from stiffer collagen fibers to extensible elastin fibers [16]. This relaxation of vascular smooth muscle tone is in part mediated by vasodilating substances from endothelium such as nitric oxide (NO). Indeed, Wilkinson *et al*. [17] and Schmitt *et al*. [18] have demonstrated that intra-arterial infusion of *N^G^*-monomethyle-L-arginine (L-NMMA), a NO synthase inhibitor, increased pulse wave velocity (PWV) of iliac artery in animals and humans, respectively, and the effect of acetylcholine, a drug that stimulate endothelial NO production, on PWV was inhibited by coinfusion of L-NMMA. Thus, although other endothelium-derived vasodilating substances such as protanoids and endothelium-derived hyperpolarizing factors may participate, increased NO production/availability plays a key role in the regulation of arterial stiffness.

Under physiological conditions, NO production is thought to be regulated by blood flow and shear stress. Dynamic exercise increases blood flow and shear stress not only in working muscles but also in non-working muscles [19]. This increase in blood flow and shear stress associated with dynamic exercise augments endothelial function by increasing NO synthase, leading to reduced arterial stiffness observed in previous studies [8, 9]. However, endothelial function declines with ageing and hypertension [20], and arterial distensibility also declines with ageing and hypertension [12]. Moreover, while sub-maximal dynamic exercise does not, maximal dynamic exercise does markedly increase oxidative stress and leads to impaired endothelium-dependent vasodilation (endothelial dysfunction) in older patients with intermittent claudication [21]. Although we did not measure oxidative stress nor endothelial function in this study, as oxidative stress increases with ageing and hypertension [22], and considering our subjects’ age and the presence of hypertension, discrepancy between the results of previous studies [8, 9] and this study may possibly be the result of excess oxidative stress associated with ageing and maximal dynamic exercise.

### Effect of 20 Weeks of Moderate Intensity Aerobic Exercise Training on Arterial Stiffness Indices

An extended period of aerobic exercise training may result in a short-term reduction of arterial stiffness and lead to a sustained reduction in stiffness observed in cross-sectional and intervention studies [4-6]. Repeated exposures of aerobic exercise may increase basal NO production and lead to the up-regulation in NO availability and other vasodilating substances [23]. Another mechanism may, as has been suggested by Joyner [24] and Tanaka *et al*. [5], simply be a result of increased deformation of blood vessels associated with exercise. The increase in BP and HR during exercise may “stretch” collagen fibers and their cross-linking occurring as a result of ageing. With the repeated exposures of this stretch-like exercise training, there may be a reduction in arterial stiffness [25]. Intrinsic stiffness of the large arteries especially in central arteries such as the aorta and carotid artery, however, has been shown to increase with ageing expressed as both β stiffness index and Young’s elastic modulus [26, 27], suggesting a progressive increase in collagen content and elastin fiber fracture in arterial walls. Furthermore, these studies have also shown that intrinsic stiffness of the large arteries between normotensives and hypertensives was not different when the same age groups were compared using an index independent of distending pressure. Indeed, subjects’ average age in the Tanaka *et al*’s study [5] was 53 yrs while our subjects’ was 68 yrs. Hence, although our subjects performed aerobic exercise training longer (20 weeks) than Tanaka *et al*’s subjects did (3 months), even 20 weeks of moderate intensity aerobic exercise training failed to modify arterial stiffness in this study due perhaps to a “larger” ageing effect and the presence of hypertension. Our results are in agreement with those of Ferrier *et al*. [10] where 8 weeks of moderate intensity aerobic exercise training did not modify arterial stiffness in older isolated systolic hypertensives (average age 64 yrs).

### Limitation

There are several limitations that need to be mentioned. A relatively small sample size and lack of control group in this study might introduce a statistical error. Second, we used brachial PP, instead of carotid PP, for obtaining arterial stiffness indices (arterial distensibility and β stiffness index) of the carotid artery, which may introduce an error in the calculation of arterial stiffness indices because of PP amplification from central to distal arteries [28]. While PP amplification becomes less with ageing and hypertension (meaning PP of both central and peripheral arteries becomes similar), it is still desirable to measure PP at the same site as diameter and thus the use of brachial PP may have overestimated carotid PP in this study. Third, continuation of drug treatment in our subjects might have affected vascular function. This is, however, less likely because drug regimens in our subjects had been stable for more than a year before the study entry and throughout the study period. Finally, because of the indication of systemic effect of aerobic exercise training on arterial stiffness [5], we did not measure an artery in the lower body such as a femoral artery even if our subjects primarily performed lower-body aerobic exercise such as walking and cycling. The influence of training mode on arterial stiffness requires further investigation.

## CONCLUSION

Our results show that, in older hypertensives, 1) acute maximal dynamic exercise had no impact on arterial stiffness of the carotid and brachial arteries, and 2) 20 weeks of moderate intensity aerobic exercise training failed to modify arterial stiffness, probably due to the “larger” ageing effect with the presence of hypertension.

## Figures and Tables

**Fig. (1):**
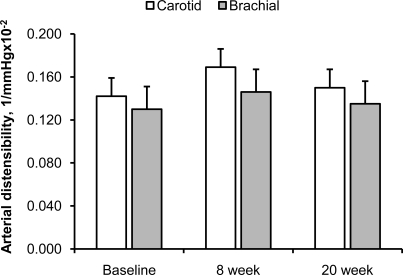
Arterial distensibility of the carotid (white bars) and brachial (gray bars) arteries following 20 weeks of aerobic exercise training. Data are means ± SE.

**Fig. (2):**
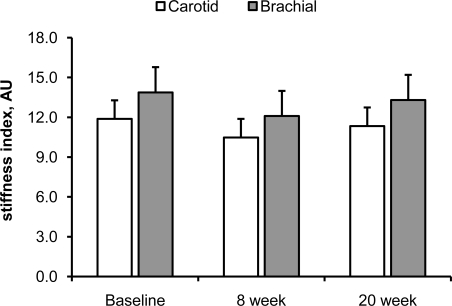
β stiffness index of the carotid (white bars) and brachial (gray bars) arteries following 20 weeks of aerobic exercise training. Data are means ± SE.

**Table 1.** Selected Subject’s Characteristics

** **	**Baseline**	**8 Week**	**20 Week**
Age, years	68.2 ± 5.4		
Height, cm	162.7 ± 7.1	-	-
Weight, kg	86.6±16.7	86.2±16.6	86.3±16.2
VO_2_max, ml/kg/min	32.2 ± 7.7	-	34.3 ± 4.9

Values are means ± SD

**Table 2.** Arterial Stiffness Indices of the Carotid and Brachial Arteries at Baseline, Immediately Following, and 24hrs Following an Acute Maximal Treadmill Exercise

** **	**Baseline**	**Post Ex**	**24h Post Ex**	**Time**	**Training**	**Interaction**
*Carotid*						
AD						
pre training	0.15 ± 0.02	0.15 ± 0.02	0.16 ± 0.01	0.65	0.50	0.48
post training	0.15 ± 0.02	0.15 ± 0.02	0.18 ± 0.01			
β						
pre training	11.9 ± 1.3	12.6 ± 1.9	11.4 ± 1.0	0.37	0.27	0.54
post training	11.3 ± 1.3	10.1 ± 1.9	9.1 ± 1.0			
*Brachial*						
AD						
pre training	0.14 ± 0.02	0.14 ± 0.02	0.15 ± 0.02	0.91	0.72	0.91
post training	0.13 ± 0.02	0.14 ± 0.02	0.14 ± 0.02			
β						
pre training	13.9 ± 2.0	14.4 ± 2.3	14.8 ± 1.7	0.97	0.50	0.59
post training	13.3 ± 2.0	12.5 ± 2.3	11.9 ± 1.7			

Values are means ± SE. AD, arterial distensibility. β, β stiffness index.Time, main effect of time (p value). Training, main effect of training (p value).Interaction, time and training interaction (p value)

**Table 3.** Blood Pressure Indices at Baseline, Immediately Following, and 24 hrs Following an Acute Maximal Treadmill Exercise

** **	**Baseline**	**Post Ex**	**24h After**	**Time**	**Training**	**Interaction**
SBP, mmHg						
pre training	154.2 ± 13.3	148.8 ± 9.3	147.6 ± 13.2	0.09	0.38	0.43
post training	146.6 ± 16.6	147.9 ± 14.6	141.1 ± 13.7			
DBP, mmHg
pre training	81.2 ± 8.5	79.6 ± 4.2	76.6 ± 7.5	0.02	0.40	0.54
post training	77.1 ± 7.0	78.4 ± 6.3	74.3 ± 7.7			
PP, mmHg						
pre training	73.3 ± 7.4	69.2 ± 8.7	71.0 ± 9.3	0.36	0.59	0.40
post training	69.4 ± 15.0	69.5 ± 11.9	66.7 ± 12.2			
MAP, mmHg						
pre training	105.7 ± 9.5	101.1 ± 6.4	96.8 ± 9.3	0.01	0.61	0.24
post training	100.2 ± 8.7	101.6 ± 8.2	96.6 ± 8.3			
HR, beats/min						
pre training	61.4 ± 24.7	74.5 ± 14.6	65.1 ± 14.8	0.00	0.81	0.86
post training	64.8 ± 13.4	75.5 ± 13.9	66.1 ± 14.3			

Values are means ± SD. SBP, systolic blood pressure. DBP, diastolic blood pressure. PP, pulse pressure. MAP, mean arterial pressure. HR, heart rate

**Table 4.** Blood Pressure Indices at Baseline, at 8 Week, and Following 20 Weeks of Aerobic Exercise Training

** **	**Baseline**	**8 Week**	**20 Week**	**Time**
SBP, mmHg	154.2 ± 13.3	142.7 ± 11.4	146.6 ± 16.6	0.17
DBP, mmHg	81.2 ± 8.5	75.1 ± 5.3	77.1 ± 7.0	0.10
PP, mmHg	73.3 ± 7.4	67.7 ± 12.8	69.4 ± 15.0	0.38
MAP, mmHg	105.7 ± 9.5	97.6 ± 5.0	100.2 ± 8.7	0.10
HR, beats/min	61.4 ± 24.7	66.8 ± 12.7	64.8 ± 13.4	0.48

Values are means ± SD. SBP, systolic blood pressure. DBP, diastolic blood pressure. PP, pulse pressure. MAP, mean arterial pressure. HR, heart rate.
